# The Effects of Chewing Gum in Preventing Eyestrain

**DOI:** 10.1155/2020/2470473

**Published:** 2020-11-16

**Authors:** Ken Asakawa, Susumu Kanno, Tomonori Ando, Kenji Osawa, Hitoshi Ishikawa

**Affiliations:** ^1^Department of Orthoptics and Visual Science, School of Allied Health Sciences, Kitasato University, Kanagawa, Japan; ^2^LOTTE Co., Ltd, Central Laboratory, Saitama, Japan

## Abstract

**Purpose:**

To investigate the effects of chewing gum and tablet candy to reduce eyestrain in healthy individuals.

**Materials and Methods:**

A double-blinded crossover trial was conducted. Forty-six healthy individuals (23 men, 23 women) between 20 and 59 years old, feeling eyestrain, were enrolled. Each 10-year age group included 12 individuals except the 30s group, which included 10 individuals. A visual task was performed on reading material displayed on a computer screen at a fixed distance for 60 min. Gum or tablet candy of two pieces were chewed for two 15-min periods starting 15 and 45 min after starting to read. Subjects chewed gum on Day 1 and tablet candy on Day 2, and vice versa. Primary outcome is as follows: subjective eye fatigue (eye tiredness, eye heaviness, blurred vision, double vision, and eye dryness) using a visual analog scale (VAS). Secondary outcomes are as follows: subjective accommodation from near and far points of accommodation measured with a D'ACOMO, spherical equivalent refraction, and eye dryness by analyzing ring break-up time (RBUT) measured with the RT-7000 Auto Ref-Topographer.

**Results:**

The VAS scores of subjective eye fatigue were not significantly changed between chewing gum and tablet candy (*P* = 0.397 − *P* = 0.909). Those scores of eye tiredness and eye heaviness were significantly longer before and after the visual task with tablet candy (*P* = 0.013 and *P* = 0.025, respectively) but not with chewing gum. The changes of subjective accommodation were significantly lower after the visual task between chewing gum and candy (*P* = 0.043). There were significant differences among each age group (20 s vs. 30 s, *P* = 0.594; 20 s vs. 40 s, *P* = 0.002; 20 s vs. 50 s, *P* = 0.002). After reading, the changes of spherical equivalent refraction did not indicate a shift toward myopia (*P* = 0.267). In the RBUT, there were no significant differences between the samples (*P* = 0.680).

**Conclusions:**

Chewing gum helps improve the ability of the eye to focus, especially in young adults.

## 1. Introduction

Advances in information technology have encouraged the widespread adoption of new electronic devices, starting with visual display terminals (VDT), then notebook computers, and mobile phones, with email functions, and most recently, smartphones, and tablets. This has created a new visual environment (e.g., Information and Communication Technology; Internet of Things) and previously unknown formats for the input and output of visual information. As a result of this transformation of the environment, accommodative insufficiency after excessive close VDT work leads to subjective symptoms of blurry vision and difficulty focusing, causing multiple instances of eyestrain. The number of people complaining of these symptoms is increasing, and methods of combatting this issue must be found [1–4]. Resolution of these problems is therefore desirable.

Although many aspects of the underlying mechanism of eyestrain remain unclear, continuous near vision places the ciliary muscles of the eye under excessive strain, reducing the ability of the eye to focus (i.e., affecting accommodative function) [5–8]. Relaxing the ciliary muscles may thus help to prevent or improve eyestrain [7, 9]. The best-known technique for this purpose is the use of a warm eye mask, which has been reported to increase blood flow to the ciliary muscles and improve the accommodative function [7]. The ciliary muscles, especially the circular Müller's fibers, which are innervated by the parasympathetic nerves, play an accommodative function.

Chewing is known to increase cerebral blood flow [10] and also actions on the autonomic nervous system (sympathetic and parasympathetic nerves) [11–13]. Our previous study showed that chewing gum increases blood flow to the eyes and also the parasympathetic nerves predominantly act to contract the iris sphincter muscle and thus reduce the diameter of the pupil [14]. The pupil is a small hole surrounded by the iris which controls its aperture by two smooth muscles, the iris dilator muscle (innervation by the sympathetic nerve) and the iris sphincter muscle (innervation by the parasympathetic nerve). Moreover, when viewing nearby objects, “near responses,” consisting of convergence, accommodation, and pupil miosis are activated, and these responses are known to be cross-linked in the cortical center [15].

In this present study, we hypothesized that chewing gum may also activate the circular Müller's fibers of the ciliary muscle, which are innervated by the parasympathetic nerves, to improve the ability of the eye to focus (accommodative function) and thus reduce the subjective eye fatigue. A warm eye mask could not do so during VDT work; however, by chewing gum, this could possibly be accomplished. Thus, we considered that chewing gum could be a simple and easy way to prevent eyestrain. We, therefore, investigated the effects of chewing gum and tablet candy to reduce eyestrain in healthy individuals who felt eyestrain while using a computer or smartphone for prolonged periods.

## 2. Materials and Methods

### 2.1. Subjects

The subjects were recruited by a contract research organization, SOUKEN CO., Ltd. (Tokyo, Japan). They received a full explanation of the study and provided written informed consent. They were asked to refrain from drinking alcohol from the day before and throughout the study and were asked not to read printed materials, other than the test matter, and not watch videos on a smartphone or other device on the day of the study.

Exclusion criteria included pregnancy or lactation, taking regular dietary supplements, allergy to foods containing gelatin (e.g., gum and tablet candy), sufficiently severe dry eye such that refraining from blinking for 5 seconds was impossible, or an inability to either chew gum or tablet candy. Thus, 4 individuals (2 men, 2 women) who had severe dry eyes were excluded from the 30s group, which had consisted of 14 individuals.

The study subjects were comprised of 46 individuals (23 men, 23 women) between 20 and 59 years old who felt eyestrain while using a computer or smartphone and who did not suffer from any eye disorders other than refractive abnormality. Each age group included 12 subjects (6 men, 6 women) except the 30 s group (5 men, 5 women); the mean ages for each group were 22.3 ± 2.7 (mean ± standard deviation), 35.5 ± 2.8, 43.8 ± 2.4, and 55.4 ± 3.0 years for subjects in the 20 s, 30 s, 40 s, and 50 s groups, respectively.

### 2.2. Study Design

This was a double-blinded crossover trial. The subjects were divided into blocks by age and sex, and stratified randomization was conducted to allocate them to undergo measurements either by first chewing the test gum followed by chewing the control tablet candy or in the opposite order. There was no carryover effect evidenced with the testing (Table 1).

This study was approved by the Research Ethics Committee of Kitasato University School of Allied Health Sciences (approval No. 2019-001). This study was registered with the UMIN clinical trials registry (UMIN000037406).

### 2.3. Blinding of Subjects

Although the actual purpose of this study was to investigate the effect of chewing gum while viewing a computer screen, subjects were told that the purpose was to investigate the effect of sample substance (gum or tablet candy) while working on a computer. The subjects did not know which type of test substance they were given. The actual purpose of the study and the reason why it could not be disclosed in advance were explained at the end of the study.

### 2.4. Blinding of Examiners

Before starting the visual task, an examiner (S. Kanno) handed the samples, wrapped up to conceal the contents. The subjects were instructed to open the package and immediately put the contents into their mouths and start chewing the sample. During the visual reading task, the other examiner (K. Asakawa) waited in a separate room from where the subjects could not be seen.

### 2.5. Testing

The tests were performed between 9 : 00 A.M. and 4 : 00 P.M. and were conducted in an examination room with a light level of approximately 500 lx, a constant temperature of 24°C, and humidity within the range of 40%–60%. Tests following the procedures described in (1)–(6) below were carried out on 2 days, then evaluated. The starting times, food, and beverage intake (water *ad libitum*), for each participant, were matched on 2 days.

Primary outcome of testing is the following: subjective eye fatigue. Secondary outcomes of testing are the following: subjective accommodation, spherical equivalent refraction, and eye dryness. The tests were performed in the same order for each subject for each eye, right eye first (except the subjective eye fatigue).

1. After arriving at the examination room, the subjects received an explanation of the study and provided written informed consent. They underwent routine visual acuity and refraction tests
2. A 30-min break was provided
3. Baseline subjective eye fatigue, subjective accommodation, spherical equivalent refraction, and eye dryness were measured
4. A visual task was performed, comprised of reading on a computer screen at a fixed distance for 60 min (with a 1-min break after the first 30 min). Subjects selected their own reading material from the free website (https://www.satokazzz.com/books/)
5. A sample substance (gum or tablet candy of two pieces) was chewed for two 15-min periods starting at 15 and 45 min after starting reading. Subjects who chewed gum on Day 1 chewed tablet candy on Day 2, and vice versa
6. Subjective eye fatigue, subjective accommodation, spherical equivalent refraction, and eye dryness were measured after the visual reading task

### 2.6. Sample Substance

Gum (1.0 g/piece) contained maltitol, gum base, mint flavor, gelatin, sugar ester, and glycerin. Tablet candy (0.7 g/piece) contained the same nutritional ingredients and flavor as the gum except for the gum base. The amount of energy and the constitution of the gum and the tablet candy were the same (carbohydrate, protein, and fat: 100, 0, and 0, respectively).

### 2.7. Primary Outcome of Testing

#### 2.7.1. Subjective Eye Fatigue

Five categories were evaluated using a visual analog scale (VAS): eye tiredness, eye heaviness, blurred vision, double vision, and eye dryness. The severity of the complaint was evaluated on a scale with 0 mm representing “not present at all” and 100 mm as “very severe.” Medical questionnaires were not used in this investigation of subjective symptoms to avoid asking leading questions, and the subjects indicated their current conditions themselves by drawing a perpendicular line across the scale for each of the five categories.

### 2.8. Secondary Outcomes of Testing

#### 2.8.1. Subjective Accommodation

This value was measured with a D'ACOMO (WOC, Kyoto, Japan). A visual target (yellow cross) was moved toward the subject at 0.2 D/s to determine the accommodation near point and away from them to determine the far point of accommodation. Distances at which the target became blurred were measured three times each (in centimeters), and amplitude of subjective accommodation (D) was calculated from the values of the near and far points of accommodation. The measurements were performed wearing a +4.0 D lens when measuring far points and when subjects could only see the blurred visual target. Subjective accommodation was then calculated as 100/near point–100/far point.

#### 2.8.2. Spherical Equivalent Refraction

Spherical and cylindrical powers were measured three times with the RT-7000 Auto Ref-Topographer (Tomey, Nagoya, Japan), and the spherical equivalent refraction diopter (D) was calculated from those values.

#### 2.8.3. Eye Dryness

This value was measured with the RT-7000 Auto Ref-Topographer and Tear Stability Analysis System (TSAS) (Tomey, Nagoya, Japan). The subject was instructed to keep the eyes open for 10 sec. Severity of eye dryness was then evaluated by measuring ring break-up time (RBUT). In accordance with the algorithm provided by Tomey, the RBUT was measured by analyzing break-up of the tear layer within a 6-mm radius of the center of the cornea and its deformation over time and measuring the number of seconds required to reach the cut-off value of –0.5 D.

### 2.9. Statistical Analyses

Statistical analyses were performed by ESUMI Co., Ltd., (Tokyo, Japan) using SPSS, version 26.0 (IBM, Armonk, NY, USA). Carryover effect of the testing was confirmed using the Paired *t*-test. Results are presented as mean ± standard deviations. Comparisons of between (*Δ*) the chewing gum and tablet candy and before and after the chewing gum or tablet candy were performed using the Wilcoxon signed-rank test. Effect size (*r*) was also calculated. Each age group was compared using the Kruskal-Wallis test, and the Mann–Whitney *U* test with Bonferroni's correction was used to determine the differences among each age group for post hoc comparisons. Results are presented as median and interquartile range (25th, 75th percentiles). *P* values of <0.05 were considered statistically significant.

## 3. Results

Table 2 shows the data of between the chewing gum and tablet candy in all the subjects, and those in each age group are shown in Table 3.  Table 4 shows the data of before and after the chewing gum or tablet candy in all the subjects.

### 3.1. Primary Outcome of Testing

#### 3.1.1. Subjective Eye Fatigue

The VAS scores of subjective eye fatigue were not significantly changed after the visual task between chewing gum and tablet candy in any of the categories (eye tiredness, *P* = 0.657; eye heaviness, *P* = 0.694; blurred vision, *P* = 0.909; double vision, *P* = 0.397; eye dryness, *P* = 0.757). There were no significant differences among any of the age groups with chewing gum and tablet candy. Compared to before the visual task, the VAS scores of eye tiredness and eye heaviness were significantly longer after the visual task with tablet candy (*P* = 0.013 and *P* = 0.025, respectively) but not with chewing gum (*P* = 0.059 and *P* = 0.075, respectively).

### 3.2. Secondary Outcomes of Testing

#### 3.2.1. Subjective Accommodation

The changes in subjective accommodation were significantly lower after the visual task between the chewing gum and tablet candy (*P* = 0.043, *r* = 0.211). There were significant differences among each age group (*P* < 0.001) (20 s vs. 30 s, *P* = 0.594, *r* = 0.249; 20 s vs. 40 s, *P* = 0.002, *r* = 0.527; 20 s vs. 50 s, *P* = 0.002, *r* = 0.513). This median value was 3.9 D and 4.0 D with chewing gum and 4.2 D and 3.8 D with tablet candy before and after the visual reading task, respectively (chewing gum, *P* = 0.579; tablet candy, *P* = 0.014).

#### 3.2.2. Spherical Equivalent Refraction

The changes of spherical equivalent refraction did not indicate a shift toward myopia (*P* = 0.267). Significant differences were obtained in each age group (*P* = 0.010). There were significant differences between the 30s and 40s groups (*P* = 0.007, *r* = 0.487). The values of before and after the visual task were -1.19 D and -1.00 D, respectively, for chewing gum (*P* = 0.361) and -1.00 D and -1.25 D, respectively, for chewing tablet candy (*P* = 0.008).

#### 3.2.3. Eye Dryness

There were no significant differences between chewing gum and tablet candy (*P* = 0.680). Before and after the visual task, the RBUT values were 10.0 sec and 9.6 sec, respectively, with chewing gum (*P* = 0.053) and 9.9 sec and 9.6 sec, respectively, with tablet candy (*P* = 0.132).

## 4. Discussion

The effects of chewing gum demonstrated that the maintenance of homeostasis, in the forms of blood pressure and heart rate, was mediated by the sympathetic and parasympathetic nerves [11–13]. Actions on the autonomic nervous system of the eye were also reported [14]. In a previous study, we found that pupil diameter contracted significantly after chewing gum compared with that at the baseline before chewing gum in young adults [14]. Whether that was due to sympathetic nerve inhibition or parasympathetic nerve activity had been unclear; however, we concluded that it was most likely due to the predominating parasympathetic nerve activity. Furthermore, previous studies have demonstrated that mastication affects human cognitive processing, including choice reaction time [16], positive mood [17], working memory [18, 19], somatosensory processing [20], motor preparation [21], and Go/No-go decision-making [22].

One factor involved in the mechanism of eye fatigue is central fatigue due to processing visual information associated with cognitive function. Subjective fatigue reflects fatigue in the cognitive processes in the central nervous system. A study of cerebral blood flow responses while fatigued found that the blood flow response decreased in associated areas of the visual cortex [23]. Conversely, the increased cerebral blood flow, as a result of chewing gum, has been suggested to help increase alertness or at least attenuate reductions in alertness [24, 25]. Regarding the findings related to relaxation caused by odor and/or taste, rather than mastication, we used samples with the same odors and tastes. Moreover, because the ability to concentrate on a visual task could be decreased during chewing gum, thereby possibly decreasing eyestrain, we checked the number of words each participant read during the chewing gum and tablet candy trials. There were no significant differences in the number of words read between the gum and tablet candy trials. We therefore considered that the gum and candy trials were carried out in as nearly the same conditions as possible, except for mastication. On the other hand, the effects of mastication may have differed among the subjects. These effects may be related to the frequency of chewing gum in daily life. There may be a relationship between the typical use of chewing gum and the changes in this study. We therefore found that there is no difference in the subjective eye fatigue between the gum and candy trials. However, before and after chewing gum, eye tiredness and eye heaviness was reduced. It is warranted to investigate this issue with subjects whose lifestyle habits, e.g., frequency of chewing gum, are the same. This could be considered a limitation in this study.

Eye fatigue is also believed to be caused by the ciliary muscles, which are involved in the accommodative function [5–8]; and warming the area around the eyes has been shown to increase blood flow and improve the accommodative function [9]. In our previous findings, chewing gum increased blood flow to the area around the eyes and caused the parasympathetic nerves predominantly act to contract the pupillary sphincter [14]. Our findings in the present study showed that chewing gum significantly reduced declines in the amplitude of subjective accommodation after the visual reading task, which may have been due to factors including improved blood flow in the eyes and activation of the Müller's ciliary muscles by the parasympathetic nerves. The clinical implication has less significance for affecting accommodative function from a little bit of effect size. However, there were significant differences among each age group, especially the 20 s group. Therefore, chewing gum helps to inhibit the declines in subjective accommodation and to improve the ability of the eye to focus by the action of the parasympathetic nerves in young adults who have sufficient accommodative function.

A previous study found that if the ciliary muscles remain in a continuous state of strain due to VDT work, transient myopia develops [26]. However, there were no significant changes in the spherical equivalent refraction after the visual task and no shift toward myopia (only 0.25 D) in the present study. Thus, fluctuations in accommodation may have been involved. “Fluctuations in accommodation” refers to the fact that even when the eye is focused on a stationary target, its refraction is not constant but exhibits slight fluctuations [27]. Because the value is around 0.3 D, the change after the visual reading task when subjects were chewing gum or tablet candy was only a slight change that was not reflected in subjective symptoms (e.g., blurry vision and difficulty focusing).

Factors reported to affect dry eye include a reduced frequency of blinking during VDT work [28] and reduced tear secretion due to increased sympathetic nervous activity as a result of stress or other causes [29], and lacrimal gland hypofunction [30]. Because tear production by the lacrimal glands is stimulated by the parasympathetic nerves [31], if chewing gum results in parasympathetic nerves predominance, tear production may also be improved. However, in the present study, there were no significant differences between each sample substance in the values of RBUT with TSAS. Tears are also derived from the meibomian glands, and meibomian secretions help stabilize the tear film [32]. One of the positive aspects is that chewing gum may help activate the lacrimal glands but not the meibomian glands, and they could conceivably also help to alleviate eye dryness. However, it is still unclear how chewing gum involves the lacrimal glands.

## 5. Conclusions

These results show that in healthy individuals, especially in young adults, who feel eyestrain, chewing gum helps to inhibit the declines in subjective accommodation and to improve the ability of the eye to focus by the action of the parasympathetic nerves. However, the mechanisms underlying eye fatigue are extremely complex, and further in-depth investigations are warranted to elucidate the changes that take place in the eye.

## Figures and Tables

**Table 1:** Reproducibility of the values before chewing gum or tablet candy (carryover effect).

Outcomes	Number of data	Difference of mean value before chewing sample	SD	95% confidence interval		*P* value
		(gum-tablet candy)		Lower limit	Upper limit	
Subjective accommodation (D)	92	-0.03	0.97	-0.23	0.17	0.786
Spherical equivalent refraction (D)		-0.03	0.29	-0.09	0.03	0.393
Ring break-up time (sec)		-0.04	3.31	-0.73	0.64	0.903

SD: standard deviation. Paired *t*-test.

**Table 2:** Data of between chewing gum and tablet candy in all subjects.

Outcomes		Number of data	Gum (*Δ*)	Tablet candy (*Δ*)	*P* value	*r*
			Median (IQR)			
VAS (mm)	Eye tiredness	46	1.5 (-6.5, 19.8)	11.5 (-7.8, 28.5)	0.657	0.065
	Eye heaviness		3.5 (-9.3, 19.5)	4.5 (-4.3, 25.3)	0.694	0.058
	Blurred vision		0 (-2.3, 15.0)	0 (-5.8, 19.3)	0.909	0.017
	Double vision		0 (-1.0, 7.3)	1.0 (-1.3, 14.5)	0.397	0.125
	Eye dryness		0 (-9.8, 16.3)	1.0 (-12.0, 19.3)	0.757	0.046
	Subjective accommodation (D)	92	-0.04 (-0.32, 0.42)	-0.14 (-0.50, 0.22)	0.043^∗^	0.211
	Spherical equivalent refraction (D)		0 (-0.25, -0.13)	0 (-0.25, 0.13)	0.267	0.116
	Ring break-up time (sec)		0 (-2.35, 0.15)	0 (-1.15, 0)	0.680	0.043

IQR: interquartile range (25th, 75th percentiles); VAS: visual analog scale. ^∗^*P* < 0.05; Wilcoxon signed-rank test. *r*: effect size.

**Table 3:** Data of between chewing gum and tablet candy in each age group.

	Outcomes		20 s (*n* = 12)	30 s (*n* = 10)	40 s (*n* = 12)	50 s (*n* = 12)	*P* value
			Median (IQR)				
Gum (*Δ*)	VAS (mm)	Eye tiredness	7.0 (-4.5, 21.3)	2.5 (-5.5, 46.3)	0 (-5.3, 11.8)	1.5 (-15.3, 23.8)	0.794
		Eye heaviness	5.0 (-9.3, 19.5)	9.5 (-4.0, 25.8)	-0.5 (-24.8, 13.0)	8.0 (-8.3, 27.3)	0.424
		Blurred vision	0 (-0.8, 6.3)	5.5 (0, 21.3)	0 (-15.8, 20.5)	-2.0 (-11.5, 11.8)	0.537
		Double vision	0 (-1.8, 5.8)	0.5 (-0.3, 16.3)	0 (0, 8.8)	0.5 (-4.0, 13.3)	0.789
		Eye dryness	3.0 (-11.3, 12.8)	17.5 (-0.3, 36.3)	-1.5 (-15.3, 10.3)	-0.5 (-17.3, 6.8)	0.240
		Subjective accommodation (D)	-0.2 (-0.9, 0.4)	-0.2 (-0.6, 0.2)	0.2 (-0.1, 0.6)	-0.1 (-0.2, 0.3)	0.055
		Spherical equivalent refraction (D)	0 (-0.1, 0.2)	0 (-0.3, 0)	-0.1 (-0.4, 0.3)	0 (-0.3, 0.1)	0.345
		Ring break-up time (sec)	-0.1 (-3.2, 0)	0 (-4.2, 0)	-0.2 (-2.8, 2.2)	0 (-1.3, 0.6)	0.417
Tablet candy (*Δ*)	VAS (mm)	Eye tiredness	14.5 (9.0, 34.3)	14.0 (-17.5, 27.5)	0.5 (-14.0, 24.3)	15.5 (-14.5, 34.8)	0.524
		Eye heaviness	3.0 (-0.8, 35.0)	4.0 (-7.8, 20.8)	-1.0 (-17.3, 10.0)	14.0 (-1.8, 28.5)	0.355
		Blurred vision	2.0 (-1.5, 18.3)	2.0 (-20.3, 31.0)	2.5 (-10.3, 17.8)	-1.5 (-9.5, 18.5)	0.792
		Double vision	0.5 (-3.3, 9.0)	0 (-1.8, 20.5)	4.0 (0, 19.5)	5.5 (-2.0, 12.0)	0.514
		Eye dryness	11.0 (-0.8, 32.5)	5.5 (-8.3, 37.0)	0 (-15.8, 10.0)	0 (-25.5, 17.5)	0.237
		Subjective accommodation (D)	-0.7 (-1.4, -0.2)	-0.2 (-0.9, 0.3)	0.1 (-0.1, 0.4)^†^	0 (-0.2, 0.2)^‡^	< 0.001^∗∗∗^
		Spherical equivalent refraction (D)	0 (-0.3, 0.1)	0 (0, 0.1)	-0.3 (-0.4, 0)^§^	0 (-0.3, 0.1)	0.010^∗∗^
		Ring break-up time (sec)	0 (-1.7, 0)	0 (-5.9, 0)	0 (-0.9, 0)	0 (-0.1, 1.4)	0.221

IQR: interquartile range (25th, 75th percentiles); VAS: visual analog scale. ^∗∗^*P* < 0.01, ^∗∗∗^*P* < 0.001; Kruskal-Wallis test. ^†^*P* = 0.002, *r* = 0.527 (20 s vs. 40 s), ^‡^*P* = 0.002, *r* = 0.513 (20 s vs. 50 s), ^§^*P* = 0.007, *r* = 0.487 (30 s vs. 40 s); Mann–Whitney *U* test with Bonferroni's correction. *r*: effect size.

**Table 4:** Data of before and after chewing gum or tablet candy in all subjects.

Sample	Outcomes		Number of data	Before	After	*P* value	*r*
				Median (IQR)			
Gum	VAS (mm)	Eye tiredness	46	29 (4, 61)	45 (20, 66)	0.059	0.278
		Eye heaviness		15 (2, 42)	28 (8, 50)	0.075	0.263
		Blurred vision		15 (1, 55)	21 (1, 54)	0.155	0.210
		Double vision		4 (0, 20)	9 (1, 44)	0.032^∗^	0.316
		Eye dryness		25 (2, 48)	32 (8, 54)	0.401	0.124
		Subjective accommodation (D)	92	3.9 (2.7, 6.4)	4.0 (2.7, 6.4)	0.579	0.058
		Spherical equivalent refraction (D)		-1.19 (-4.09, -0.25)	-1.00 (-4.34, -0.25)	0.361	0.095
		Ring break-up time (sec)		10.0 (4.7, 10.0)	9.6 (3.4, 10.0)	0.053	0.202
Tablet candy	VAS (mm)	Eye tiredness	46	24 (4, 61)	45 (22, 63)	0.013^∗^	0.366
		Eye heaviness		17 (4, 47)	32 (9, 58)	0.025^∗^	0.330
		Blurred vision		12 (1, 46)	29 (4, 47)	0.159	0.208
		Double vision		3 (1, 20)	12 (1, 34)	0.009^∗∗^	0.384
		Eye dryness		28 (7, 58)	42 (4, 54)	0.237	0.174
		Subjective accommodation (D)	92	4.2 (2.7, 6.3)	3.8 (2.5, 6.0)	0.014^∗^	0.256
		Spherical equivalent refraction (D)		-1.00 (-3.97, -0.38)	-1.25 (-4.34, -0.38)	0.008^∗∗^	0.277
		Ring break-up time (sec)		9.9 (5.3, 10.0)	9.6 (4.3, 10.0)	0.132	0.157

IQR: interquartile range (25th, 75th percentiles); VAS: visual analog scale. ^∗^*P* < 0.05, ^∗∗^*P* < 0.01; Wilcoxon signed-rank test. *r*: effect size.

## Data Availability

The data used to support the findings of this study are included within the article.
